# miR-146a-3p suppressed the differentiation of hAMSCs into Schwann cells via inhibiting the expression of ERBB2

**DOI:** 10.1007/s00441-020-03320-8

**Published:** 2021-01-15

**Authors:** Wei Chen, Linlin Ji, Zairong Wei, Chenglan Yang, Shusen Chang, Yucheng Zhang, Kaiyu Nie, Lingli Jiang, Yurong Deng

**Affiliations:** Department of Burns and Plastic Surgery, Affiliated Hospital of Zunyi Medical College, 149 Dalian Road, Guizhou Zunyi, China

**Keywords:** Human amniotic mesenchymal stem cells, Schwann cells, miR-146a-3p, ERBB2, Neurotrophic factors

## Abstract

Human amniotic mesenchymal stem cells (hAMSCs) can be differentiated into Schwann-cell-like cells (SCLCs) in vitro. However, the underlying mechanism of cell differentiation remains unclear. In this study, we explored the phenotype and multipotency of hAMSCs, which were differentiated into SCLCs, and the expression of nerve repair-related Schwann markers, such as S100 calcium binding protein B (S-100), TNF receptor superfamily member 1B (P75), and glial fibrillary acidic protein (GFAP) were observed to be significantly increased. The secreted functional neurotrophic factors, like brain-derived neurotrophic factor (BDNF), nerve growth factor (NGF), and neurotrophin-3 (NT-3), were determined and also increased with the differentiation time. Moreover, miR-146a-3p, which significantly decreased during the differentiation of hAMSCs into SCLCs, was selected by miRNA-sequence analysis. Further molecular mechanism studies showed that Erb-B2 receptor tyrosine kinase 2 (ERBB2) was an effective target of miR-146a-3p and that miR-146a-3p down-regulated ERBB2 expression by binding to the 3′-UTR of ERBB2. The expression of miR-146a-3p markedly decreased, while the mRNA levels of ERBB2 increased with the differentiation time. The results showed that down-regulating miR-146a-3p could promote SC lineage differentiation and suggested that miR-146a-3p negatively regulated the Schwann-like phenotype differentiation of hAMSCs by targeting ERBB2. The results will be helpful to establish a deeper understanding of the underlying mechanisms and find novel strategies for cell therapy.

## Introduction

Schwann cells (SCs) play a key role in successful nerve repair and regeneration by supporting both axonal growth and myelination. The functions of SCs are myelin-sheath formation, nerve impulse transmission, and the secretion of a variety of neurotrophic factors, such as brain-derived neurotrophic factor (BDNF), glial cell-derived neurotrophic factor (GDNF), nerve growth factor (NGF), and neurotrophin-3 (NT-3), and of extracellular matrix components that provide a beneficial microenvironment for neuronal survival and axonal growth (Carr and Johnston 2017). However, the acquisition of human Schwann cell sources encounters some difficulties, such as their slow proliferation rate and limited in vitro culture. In clinical operations, autologous acquisition of SCs can cause peripheral nerve injury, but, at the same time, it also faces the problem of the lack of donors (Armaiz and Wang 2017; Jiang et al. 2017).

Adult stem cells, such as human amniotic mesenchymal stem cells (hAMSCs), which are derived from the amniotic membrane and amniotic fluid, have a great capacity for proliferation and differentiation and do not form teratomas when transplanted into animal models (Carbone et al. 2014; Diaz-Prado et al. 2011; Zheng et al. 2008). Recently, it has reported that Schwann cell-like cells (SCLCs) can be differentiated from hAMSCs (Jiang et al. 2010). However, the mechanism of differentiation has not been fully elucidated. Thus, the understanding of the molecular mechanism of the differentiation of SCLCs from hAMSCs might help to create more desirable procedures to obtain SCLC-derived lineages in vitro.

MicroRNAs (miRNAs) are a subset of RNAs that are short, non-coding RNAs of approximately 25 nucleotides, which have been shown to play multiple roles in the regulation of development and differentiation. Increasing evidences have confirmed that miRNAs participate in the differentiation of all three germ layers, including adipogenesis, osteogenesis, and myogenesis (Marti et al. 2016; Mok et al. 2017). Several miRNAs were demonstrated to be involved in the modulation of hAMSC differentiation: miR-125b positively regulated adipogenic differentiation by targeting PTEN, cyclin E1, and CDK6; miR-204 inhibited osteoblast differentiation from hAMSCs; miR-138/miR-222 over-expression contributed to hAMSC differentiation into adipocytes in pregnancy (Avendaño-Féli et al. 2019; Glemžaitė and Navakauskienė 2016; Nardelli et al. 2017; Trohatou et al. 2014, 2017). Nevertheless, seldom comprehensive analysis has been performed on the miRNAs involved in the differentiation of hAMSCs into SCLCs. Therefore, the miRNAs playing a role in the regulation and the mechanisms by which miRNAs regulate Schwann cell differentiation need to be further studied.

In this study, hAMSCs were cultured and differentiated into SCLCs, and the expression of the nerve repair-related Schwann markers S100 calcium-binding protein B (S-100), TNF receptor superfamily member 1B(P75), and glial fibrillary acidic protein (GFAP) were observed to be significantly increased. The secreted functional neurotrophic factors BDNF, NGF, and NT3 were determined and were also observed to increase with the differentiation time. Then, the expression of miRNAs was profiled during hAMSC differentiation into Schwann cell-like cells, and miR-146a-3p was significantly decreased, which might indicate that it played a negative regulatory role in SCLC lineage differentiation. Furthermore, Erb-B2 receptor tyrosine kinase 2 (ERBB2), an important regulator of the SC lineage differentiated from hAMSCs, was identified as a direct target of miR-146a-3p. The results showed that the down-regulation of miR-146a-3p would promote SC lineage differentiation and suggested that miR-146a-3p negatively regulated the Schwann-like phenotype differentiation of hAMSCs by targeting ERBB2. The results will be helpful to establish a deeper understanding of the underlying mechanisms and find novel strategies for cell therapy.

## Materials and methods

### Culture and differentiation of hAMSCs

hAMSCs were purchased from SALIAI Stem Cell Technology (Guangzhou, China). The cells were cultured in LG-DMEM (Gibco, Grand Island, USA) medium (containing 10% FBS, 2 mM glutamine, 1% non-essential amino acids, 55 mM 2-mercaptoethanol, 1 mM sodium pyruvate, 100 U/mL penicillin, and 100 mg/mL streptomycin) and inoculated in 6-well culture plates at a cell density of 5 × 10^5^/mL, at 37 °C with 5% CO_2_. After the cell confluence reached 80–90%, the cells were digested with 0.25% trypsin-0.02% EDTA solution, at 37 °C for 2–3 min. The effect of trypsin was terminated by adding culture medium. After centrifugation for 5 min, the supernatant was discarded, and the cell pellet was resuspended in culture medium, the cells were sub-cultured at a cell density of 1 × 10^7^/mL. hAMSCs at passage 3 (P3) were used in the differentiation experiments. MiR-146a-3p mimic and its negative control, and anti-miR-146a-3p inhibitor, ERBB2 siRNA and control siRNA were purchased from RiboBio (Guangzhou, China) and transfected with ribo FECT™ CP Transfection Kit (RiboBio, Guangzhou, China). All experiments and procedures were approved by the Ethics Committee of the Zunyi Medical University.

### Multipotential differentiation

For adipogenic differentiation, hAMSCs were cultured in fat-inducing medium (Iscove’s modified Dulbecco’s media (IMDM) supplemented with 10% FBS, 10 µg/mL insulin, 1 µM dexamethasone, 0.5 mM IBMX, and 0.1 mM indomethacin) for 12 days. After lipid droplets were formed, oil red O staining was performed. Cells were washed three times with PBS and fixed for 20 min in 10% neutral formaldehyde. After washing twice with PBS, oil red O buffer (Millipore, Burlington, USA) was added for 30 min, after which cells were washed again twice. Cells were, then, subjected to hematoxylin staining for 1 min. The decontamination solution was discarded, and the cells were photographed using an inverted microscope.

For osteogenic differentiation, hAMSCs were cultured with osteogenic induction medium (IMDM supplemented with 10% FBS, 5 µg/mL insulin, 0.1 µM dexamethasone, 0.2 mM vitamin C, and 10 mM beta-glycerophosphate) for 15 days. Alizarin red S (ARS) staining was performed after the formation of mineralized nodules. Cells were washed three times with PBS, fixed for 20 min in 10% neutral formaldehyde, and washed again twice with PBS. Cells were submerged in alizarin red S staining solution (Millipore, Burlington, USA) and exposed to sunlight for 30 min. After washing twice with PBS, mineralized nodules were observed and photographed using an inverted microscope.

For SCLC differentiation, hAMSCs were seeded into 6-well plates in regular culture medium. On the following day, cells were changed to differentiation basic medium DMEM/F12 contained 1 mmol/L β-mercaptoethanol on the first day. After 24 h, the medium was changed into DMEM/F12 with 35 ng/mL ATRA and 10% FBS for 3 days. After that, the medium was discarded, the cells were washed three times with PBS and changed with medium DMEM/F12 with 5 ng/ml PDGF-AA (Peprotech, USA), 10 ng/ml bFGF (Peprotech, USA), 14 lM forskolin (Sigma, USA), and 200 ng/ml heregulin (Peprotech, USA). Thereafter, the medium was changed twice a week. Cells were cleaned twice with PBS and photographed under inverted microscope at day 0, day 7, day 14, and day 21.

### MiRNA-seq analysis

To identify SCLC-related miRNAs, hAMSCs were exposed to SCLC induction medium, as described above, and total RNA was isolated using the TRIzol reagent (Invitrogen, Thermo Fisher Scientific, USA) at day 14. Sample processing, hybridization, and microRNA sequencing analysis were conducted by Majorbio Biotechnology (Shanghai, China). Briefly, a fold change ≥ 2 or ≤ 0.5 between hAMSC and induced Schwann cells samples and a *p* value < 0.05 were chosen as the cutoff criteria for differentially expressed miRNAs. Down-stream targets of miRNAs were predicted by Targetscan 7.2 (https://www.targetscan.org/vert72/), miRTarbase (https://mirtarbase.mbc.nctu.edu.tw/), and DIANA TOOLS (https://diana.imis.athena-innovation.gr/DianaTools/). Overlapping predicted targets of the top 10 differentially expressed miRNAs in different times were used in pathway enrichment analyses performed by using Metascape (https://www.metascape.org/).

### Flow cytometry

Flow cytometry was used to identify the purity of P3 hAMSCs. Cultured cells were incubated with a primary antibody against CD44, CD90, CD73, or CD105 (Abcam, Boston, USA), for 1 h on ice, to confirm the expression of the cell surface biomarkers. After washing with PBS, cells were incubated with the fluorescent dye-conjugated Alexa-488 secondary antibody (Bio-Rad, Hercules, USA), for 30 min at 4 °C. After that, cells were washed and stained with RNase and propidium iodide (PI), for 30 min, to calculate the cell proportion in each cell cycle phase. Cells were analyzed using a FACSCalibur flow cytometer with the Cell Quest acquisition software (BD Biosciences, Franklin Lakes, USA). List mode files were analyzed with the FlowJo software (Tree Star, San Carlos, USA).

### Immunofluorescence

To study the morphological changes of hAMSCs after differentiation, cells were washed three times with PBS and fixed in 4% cold paraformaldehyde for 15 min. Cells were, then, incubated in 0.2% Triton X-100, for 10 min, and blocked with 10% goat serum, for 60 min at 37 °C. Cells were incubated overnight at 4 °C with the following primary antibodies (all from Abcam, Boston, USA): rabbit anti-GFAP (1:1500), rabbit anti-P75 (1:500), and mouse anti-S100 (1:300). Cells were, then, incubated with Alexa Fluor 594- or Alexa Fluor 488-conjugated anti-mouse or anti-rabbit (1:300, Invitrogen, Carlsbad, USA) secondary antibodies. Finally, the cells were stained with DAPI/PI for 5 min. All images were photographed using a fluorescence microscope.

### Quantitative real-time quantitative PCR

Total RNA was extracted with TRIzol reagent and converted into cDNA using M-MLV cDNA kit (Invitrogen, Carlsbad, USA). The *Homo sapiens* (has)-miR-146a-3p RNA mimic and inhibitor, ERBB2 siRNAs, and control siRNAs were reverse-transcribed using a specific RT primer (RiboBio, Guangzhou, China), according to the manufacturer’s protocol. GAPDH was used as an endogenous normalization control for mRNAs, and U6 was used as endogenous normalization control for miR-146a-3p. Primer pairs for all mRNAs and miRNAs were designed using the RiboBio primer online design tool (RiboBio, Guangzhou, China). RT-qPCR was performed using the SYBR Green PCR Kit (Toyobo, Osaka, Japan) and the Applied Biosystems 7500 Real-Time PCR Detection System (Life Technologies, Carlsbad, USA). The data were analyzed using the 2^−ΔΔCt^ relative expression method. All experiments were repeated three times. Primer sequences are shown in Table 1.

**Table 1:** Primer sequence for RT-qPCR

Target gene	Direction	Sequence (5′–3′)
S-100	Forward	GGAAATCAAAGAGCAGGAGGT
	Reverse	ATTAGCTACAACACGGCTGGA
GFAP	Forward	CCTCTCCCTGGCTCGAATG
	Reverse	GGAAGCGAACCTTCTCGATGTA
P75	Forward	GGAAGCGAACCTTCTCGATGTA
	Reverse	TGAAGGCTATGTAGGCCACAA
ERBB2	Forward	GACAACTAGTACCAGAAGGCCAAGTCCGCA
	Reverse	GACAAAGCTTAGCTGTTTTCCAAAATATAT
GAPDH	Forward	TCAAGAAGGTGGTGAAGCAG
	Reverse	CGTCAAAGGTGGAGGAGTG
miR-146a-3p	Forward	CAGCCTCTGAAATTCAGTTCT
	Reverse	TCCAGTTTTTTTTTTTTTTTCTGAAGA

*RT-qPCR* reverse transcription quantitative polymerase chain reaction, *S-100* S100 calcium-binding protein B, *P75* TNF receptor superfamily member 1B, *GFAP* glial fibrillary acidic protein, *ERBB2* Erb-B2 receptor tyrosine kinase 2, *GAPDH* glyceraldehyde-3-phosphate dehydrogenase

### Western blot analysis

Total proteins were extracted from cells and tissues using RIPA solution (Beyotime, Shanghai, China), according to the manufacturer’s instructions. Equivalent amounts of proteins from each sample were separated by 10% SDS-PAGE, transferred to 0.22 μm PVDF membranes (Millipore, Burlington, USA), blocked in 5% fat-free milk for 1 h, and incubated with specific primary antibodies (all from Abcam, Boston, USA) as listed: GFAP (mouse monoclonal, 1:500), S100 (rabbit monoclonal, 1:1,000), p75NTR (rabbit polyclonal, 1:500). After the incubation with the primary antibodies, the membranes were incubated with HRP-conjugated IgG for 2 h, followed by detection with an enhanced chemiluminescence system. A GAPDH antibody (1:500) was used as control. The experiment was performed in triplicate.

### ELISA assay

The supernatants of the third generation of hAMSCs and neuron-like cells at days 3, 7, 14, and 21 after differentiation were collected, and the levels of BDNF, NGF, and NT3 in the medium from different groups were quantified using ELISA kits (Cusabio Technology, Wuhan, China), according to the manufacturer’s instructions.

### Statistical analysis

All data were expressed as the mean ± standard deviation (SD) of the mean. Data were analyzed by one-way analysis of variance (ANOVA). In comparisons involving ≥ d3 groups, one-way ANOVA followed by Bonferroni Dunn tests was used. A *p* value < 0.05 was considered statistically significant in two-tailed ANOVA.

## Results

### Characterization of hAMSCs and detection of osteogenic and adipogenic abilities of hAMSC

**Fig. 1:**
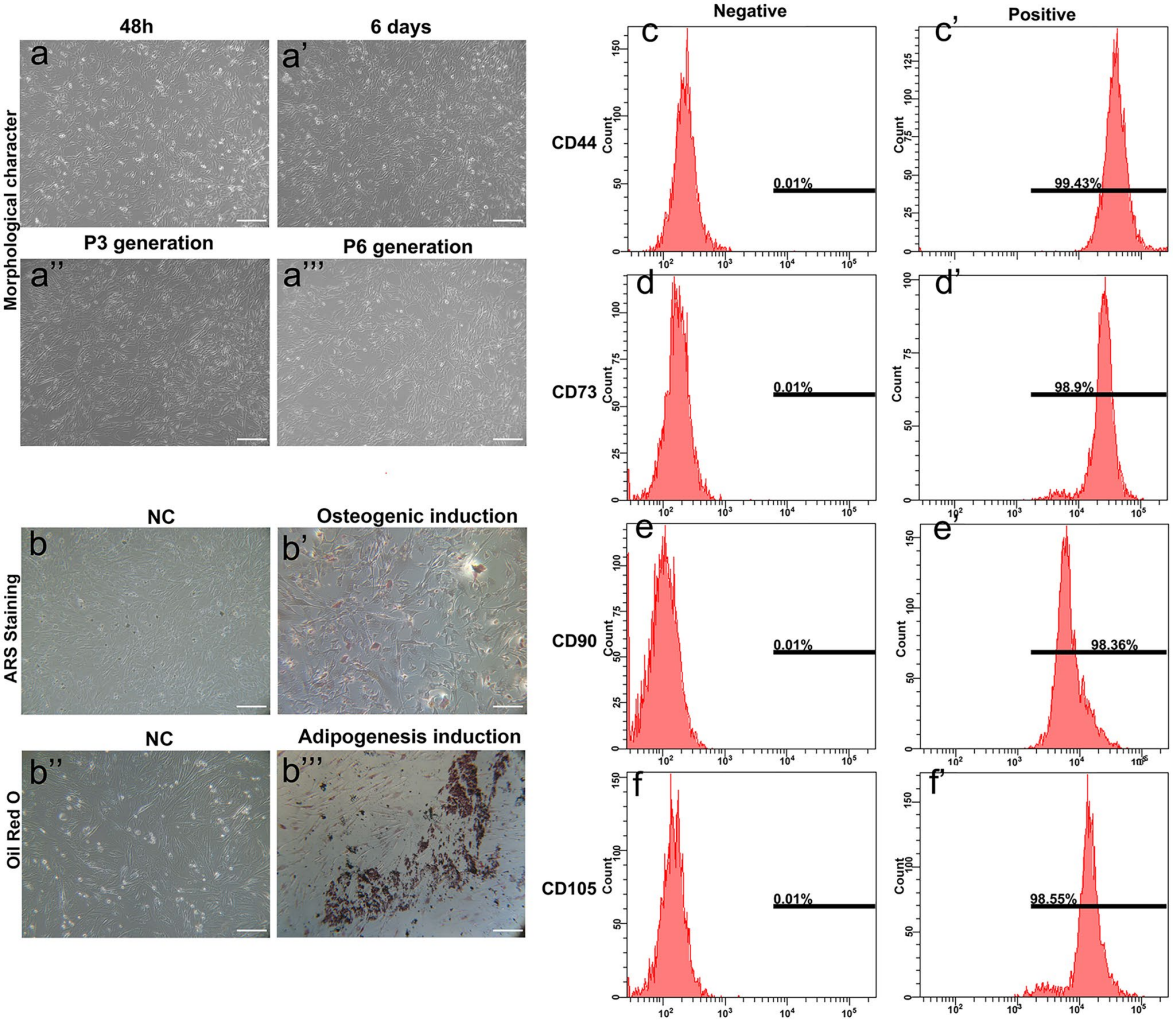
Characterization of hAMSCs and detection of osteogenic and adipogenic abilities of hAMSCs. (a) The culture and morphological identification of hAMSCs (scale bar 100 μm). The morphological characters of hAMSCs of 48 h, day 6, P3 generation and P6 generation. (b) Alizarin red S (ARS) and oil red O staining were used to detect the osteogenic and adipogenic differentiation of hAMSCs (scale bar 100 μm). (c–f) The expression of hAMSCs marker genes in P3 generation hAMSCs investigated by flow cytometry. CD44 (c), CD73 (d), CD90 (e), and CD105 (f) were all positive

HAMSCs were cultured in Schwann cells induction medium in vitro. After 48 h of hAMSC culture, the cells grew mostly oval and triangular in shape. After 6 days in culture, the cells could proliferate and be passaged successfully. After passaging, cells, especially the third-generation ones (P3), grew rapidly with high purity and aligned tightly, presenting a fibroblast-like shape as previous paper described (Romani et al. 2015; Wang et al. 2020). The sixth-generation (P6) cells grew in a spiral colony, and their morphology changed clearly to a flat shape (Fig. 1a). Therefore, P3 hAMSCs were used for further research.

CD44, CD73, CD90, and CD105 are specific markers of hAMSCs (Dominici et al. 2006; Koik et al. 2014). Therefore, hAMSCs were clearly identified by detecting the expression of the mentioned markers through flow cytometry. Differentiated cells that did not express CD44, CD73, CD90, and CD105 were used as negative controls. Results showed that CD44, CD73, CD90, and CD105 were all highly expressed (> 90%) in cultured hAMSCs (Fig. 1c–f). After 21 days of in vitro induction, the differentiation capability of the hAMSCs was detected with Alizarin red S and oil red O. hAMSCs grew from long spindle shapes into polygons or triangles in morphology, and Alizarin red S staining was positive, showing obvious osteogenic differentiation. After in vitro induction with adipogenic medium, red lipid droplets could be seen after oil red O staining in induced hAMSCs (Fig. 1b). The above results suggested that hAMSCs had good stemness and differentiation potential.

### The expression of S-100, P75, and GFAP were significantly increased after differentiation into SCLCs, and BDNF, NGF and NT3 expressions also markedly increased during differentiation

**Fig. 2:**
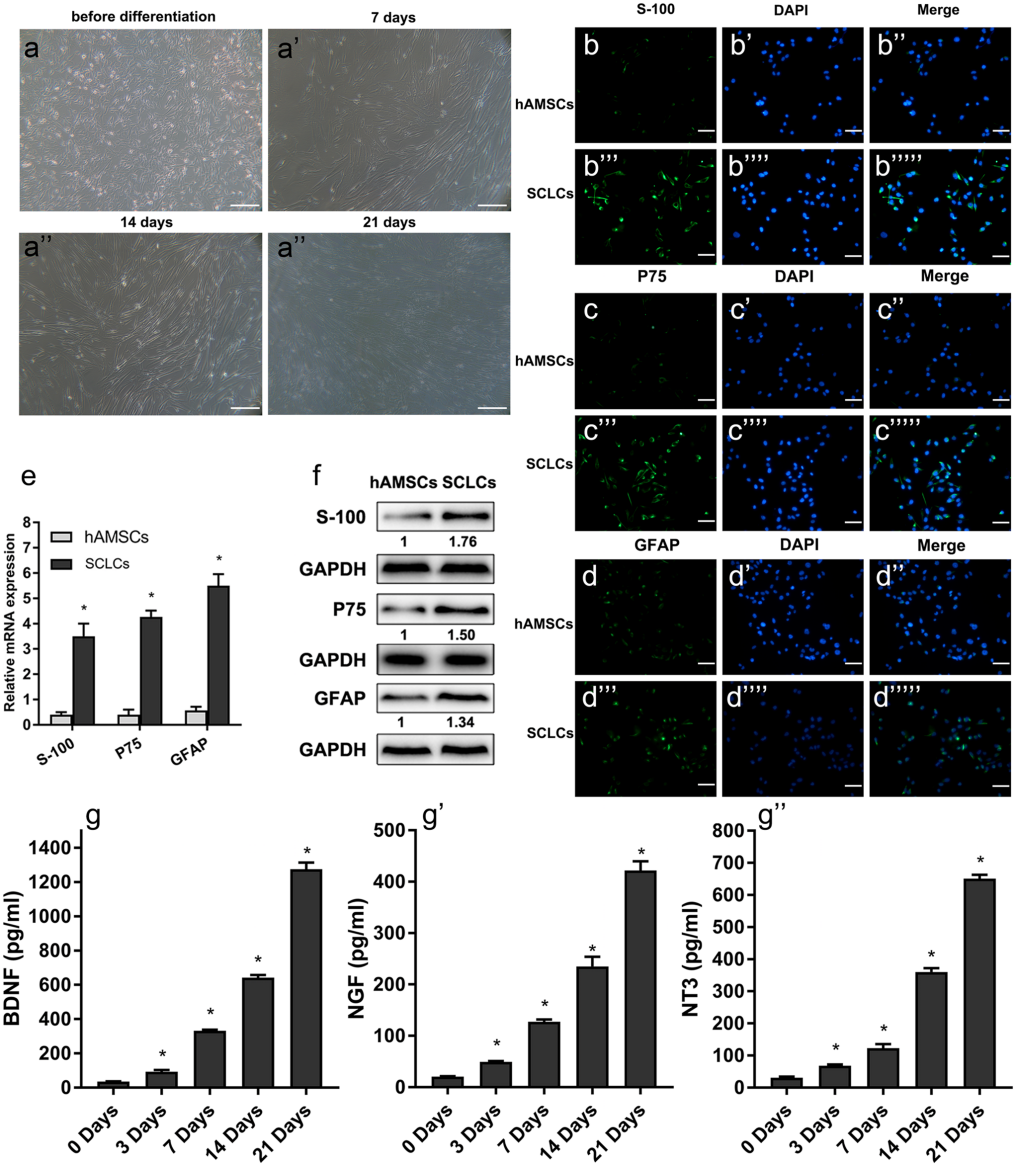
The expression of S-100, P75, and GFAP was significantly increased after differentiation from hAMSCs into SCLCs and the expression of BDNF, NGF, and NT3 also markedly increased during differentiation. (a) The culture and morphological identification of SCLCs at day 0 (before differentiation), day 7, day 14, and day 21 after differentiation induction (scale bar 100 μm). (b–d) Immunofluorescence detection expression of S-100 (b), P75 (c), and GFAP (d) in undifferentiated hAMSCs or SCLCs after differentiation for 21 days. The undifferentiated hAMSC group was used as the negative control. The fluoroscopic time was set to 500 ms. S-100, P75, and GFAP were located in the cytoplasm of SCLCs (scale bar 100 μm). (e) Detection of S-100, P75, and GFAP expression in undifferentiated hAMSCs or SCLCs after differentiation for 21 days by qPCR. (f) Detection of S-100, P75, and GFAP expression in undifferentiated hAMSCs or SCLCs after differentiation for 21 days by western blot. (g) Detection of BDNF, NGF, and NT3 level in supernatant during induction by ELISA at day 0, day 3, day 14, and day 21. Data represent mean ± S.D. *P < 0.05

The hAMSCs with high purity and uniform shapes were used for Schwann-like cell differentiation. After 7 days of differentiation, the cells adhered to the wall and grew mostly in a strip shape. After 14 days of differentiation, the strip shape of cells was more obvious, and their growth rate was slower. At day 21 after differentiation, the cells were single, flat, and slow growing (Fig. 2a).

In order to determine whether the hAMSCs had the differentiation potential to neuronal-like cells, the presence of the neurocyte-specific proteins S-100, P75, and GFAP was assessed in SCLCs 21 days after induction by immunofluorescence. The results showed that S-100, P75, and GFAP were more abundantly expressed in SCLCs after induction than in hAMSCs before induction (Fig. 2b–d). The mRNA and protein levels of S-100, P75, and GFAP were also investigated to further confirm the above results. Results from qPCR and western bolt showed that the expressions of S-100, P75, and GFAP in cells were significantly increased after differentiation (*p* < 0.05) (Fig. 2 e and f). These results indicated that hAMSCs could be differentiated into iSCs successfully.

During the differentiation from hAMSCs into SCLCs, the supernatants were collected, and the levels of BDNF, NGF, and NT3 in the supernatants were determined. The levels of BDNF, NGF, and NT3 in the supernatant increased significantly at day 3 and increased with the prolongation of the induction time (*p* < 0.05) (Fig. 2g). From the results above, we could see that BDNF, NGF, and NT3 were secreted continuously during the induction of hAMSCs into SCLCs and that their secretion increased with the prolongation of induction time (*p* < 0.05).

### MiRNA sequencing and functional analysis of differentially expressed miRNAs during the differentiation from hAMSCs into SCLCs

**Fig. 3:**
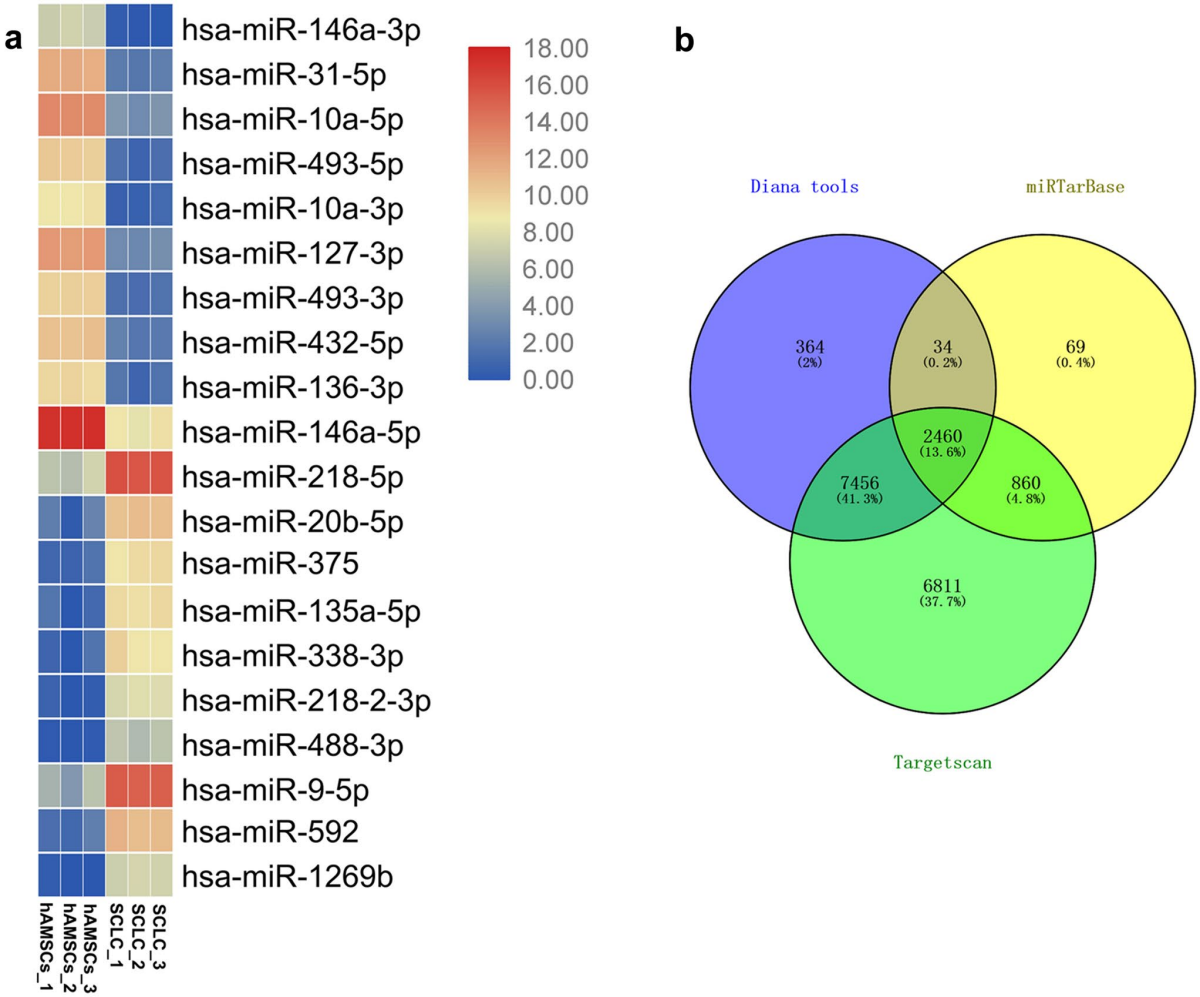
MiRNA sequencing and functional analysis of differentially expressed miRNAs during the differentiation from hAMSCs into SCLCs. **a** Heatmap of TOP 20 differentially expressed miRNAs before and after SCLC differentiation for 14 days by miRNA-seq analysis.** b** Overlapped predicted targets of miR-146a-3p of miRNA databases

**Fig. 4.**
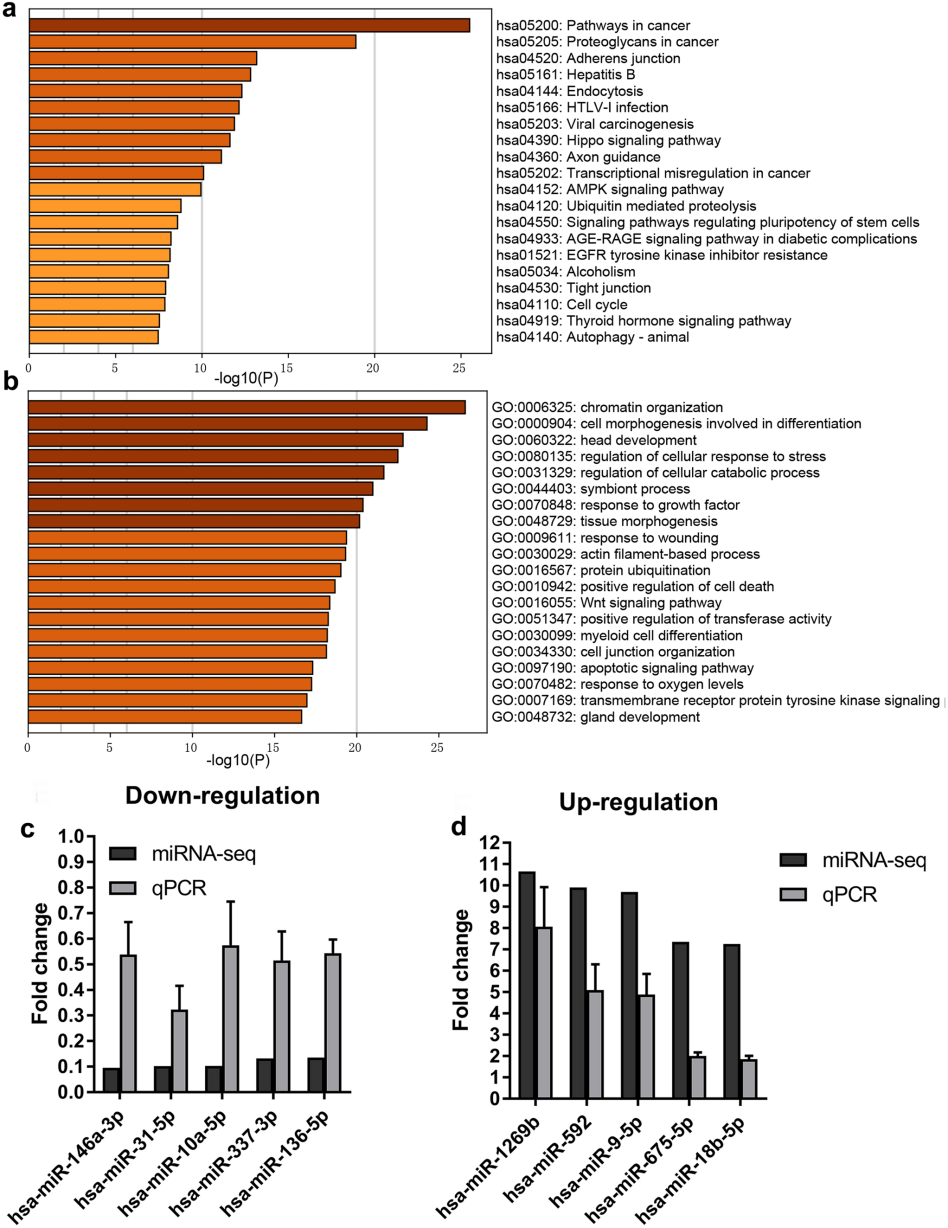
**a** KEGG pathway enrichment analysis of differentially expressed microRNAs before and after SCLC differentiation at day 14. **b** Gene ontology analysis of differentially expressed microRNAs before and after SCLC differentiation at day 14. **c**, **d** Verification of top 10 differential expressed miR-seq results by qPCR the fold changes shows the fold change of selected miRNAs between the hAMSCs and SCLC differentiation for 14 days. Data represent mean ± S.D. *P < 0.05

Based on cutoff criteria of absolute value of logFC ≥ 1 and *p* value < 0.05, 443 differentially expressed miRNAs were obtained in the miR-seq experiment. The top 20 (top 10 up-regulated and top 10 down-regulated) miRNAs with the highest |log_2_FC| values were selected as SCLCs-related miRNAs (Fig. 3a). Through the prediction of miRNA-RNA databases (DIANA TOOLS, miRTarBase and Targetscan), 2460 common target genes of the top 20 SCLCs-related miRNAs were found (Fig. 3b). According to the prediction of selected target genes of the top 20 miRNAs, Kyoto Encyclopedia of Genes and Genomes (KEGG) and Gene Ontology (GO) enrichment analysis were performed by Metascape. Top 20 GO biological process terms and KEGG pathways with significant changes before and after induction are shown in Fig. 4 a and b. KEGG and GO enrichment analysis demonstrated that the top 20 miRNAs implicated in cancer development, cell junction, and more importantly, cell differentiation related pathway, such as cell morphogenesis involved in differentiation and Wnt signalling pathway, Hippo signaling pathway signaling, and pathway regulating pluripotency of stem cells. Next, the top 10 miRNAs (top 5 up-regulated and top 5 down-regulated) were selected to verify the expression before and after induction by qPCR. The expression trend of the selected miRNAs was consistent with that of the sequencing results (Fig. 4 c and d), indicating that the sequencing results of miRNAs were reliable and could be used for further experiments.

### ERBB2 is the direct downstream target of mir-146a-3p, which regulated the expression of S-100, P75, and GFAP in cells and the secretion of BDNF, NGF, and NT3

**Fig. 5:**
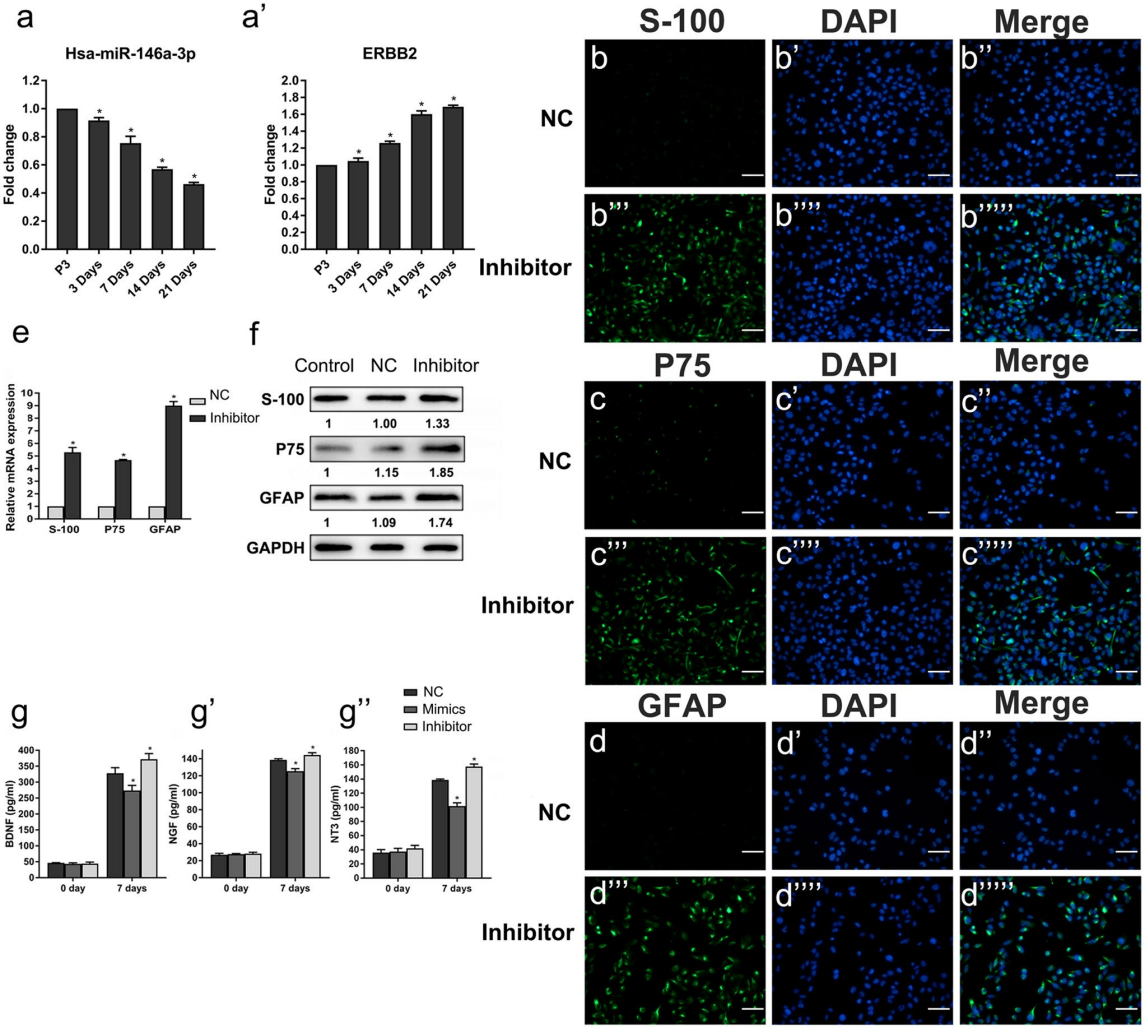
ERBB2 is the direct downstream target of mir-146a-3p, which regulated the expression of S-100, P75, and GFAP and the secretion of BDNF, NGF, and NT3 in the cells. SCLCs were divided into three groups, SCLCs without transfection (control group), SCLCs transfected with negative control sequence (NC group), SCLCs transfected with inhibitor of miR-146a-3p (inhibitor group) SCLCs transfected with miR-146a-3p mimics (mimics group). (a) Changes of the expressions of miR-146a-3p and ERBB2 during Schwann-like induction. (b–d) Immunofluorescence detection of the effect of miR-146a-3p on the expression of S-100 (b), P75 (c), and GFAP (d) of SCLCs after the differentiation at day 14. SCLCs transfected with negative control sequence was used as the negative control. The fluoroscopic time was set to 500 ms. S-100, P75, and GFAP were located in the cytoplasm of SCLCs (scale bar 100 μm). (e) Detection of the effect of miR-146a-3p on the expression of S-100, P75, and GFAP in NC group and inhibitor group at day 14 by qPCR. (f) Detection of the effect of miR-146a-3p on the expression of S-100, P75, and GFAP in NC group and inhibitor group at day 14 by western blot. (g) ELISA detection of the effect of miR-146a-3p on the expression of BDNF, NGF, and NT3 in three groups. Data represent mean ± S.D. *P < 0.05

Combining the literature with the above analysis results, we selected the miRNA that showed the largest downregulation during the differentiation, miR-146a-3p as the research target. MiRNA target prediction study showed that an important differentiation regulator ERBB2 may be the target of miR-146A-3p by binding to the 3′-UTR of ERBB2. Then, we hypothesized that miR-146a-3p regulated the differentiation from hAMSCs into SCLCs via targeting ERBB2. In order to confirm the hypothesis, cells were collected at days 0, 7, 14, and 21 during the differentiation of hAMSCs into SCLCs. The expression of miR-146a-3p and EBBB2 were measured by qPCR. The result showed that the levels of miR-146a-3p decreased and those of ERBB2 increased with the increase of the induction time and that there was a negative correlation between them (Fig. 5a). The result suggested that during differentiation, miR-146a-3p might play negatively regulated role during differentiation. The downregulation of miR-146a-3p might release its restoration on downstream target ERBB2, and cell differentiation was promoted by the upregulation of ERBB2.

MiR-146a-3p mimics and inhibitors were transfected into hAMSCs before Schwann cell differentiation for 7 days to further study the function of miR-146a-3p. The immunofluorescence analyses suggested that after inhibiting miR-146a-3p, the expression of S-100, P75, and GFAP were significantly higher than those in the control groups (Fig. 5b–d). The results from qPCR and western bolt also confirmed the finding (Fig. 4 e and f). The results above showed that transfection of miR-146a-3p inhibitors could induce the up-regulation of neurocyte-specific proteins and promote differentiation from hAMSCs into SCLCs.

The levels of BDNF, NGF, and NT3 in the supernatant of SCLCs after miR-146a-3p over-expression or interference for 7 days were measured by ELISA. The results showed that the secretion of neurotrophic factors increased after over-expressing miR-146a-3p and decreased after interfering with miR-146a-3p (Fig. 5g). This experiment suggested that miR-146a-3p might regulate the secretion of neurotrophic factors.

**Fig. 6:**
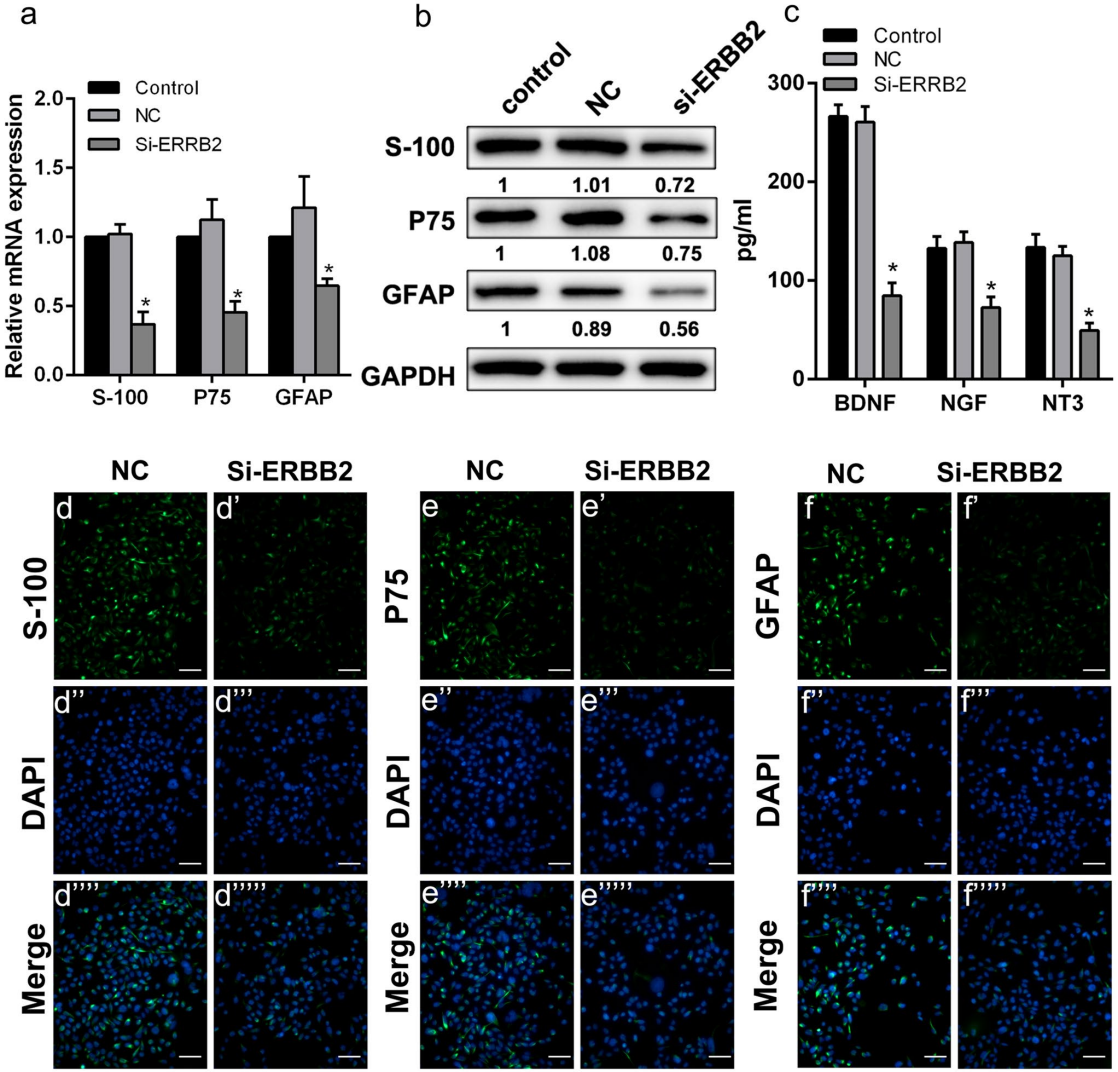
ERBB2 regulated the expression of S-100, P75, and GFAP and the secretion of BDNF, NGF, and NT3 in the SCLCs cells. SCLCs cells were divided into three groups: SCLCs without transfection (control group), SCLCs transfected with negative control sequence (NC group), SCLCs transfected with siRNA of ERBB2 (Si-ERBB2 group). (a) Detection of the effect of ERBB2 on the expression of S-100, P75, and GFAP by qPCR in three groups at day 7. (b) Detection of the effect of ERBB2 on the expression of S-100, P75 and GFAP by western blot at day 7 in three groups. (c) ELISA detection of the effect of ERBB2 on the expression of BDNF, NGF, and NT3 in three groups. (d–f) Immunofluorescence detection of the effect of ERBB2 on the expression of S-100, P75, and GFAP SCLCs transfected with negative control sequence was used as the negative control, the fluoroscopic time was set to 500 ms. S-100, P75, and GFAP were located in the cytoplasm of SCLCs (scale bar 100 μm). Data represent mean ± S.D. *P < 0.05

Furthermore, to verify the functions of ERBB2, siRNAs of ERBB2 were transfected into hAMSCs before Schwann-like cell differentiation for 7 days. The qPCR, western blot, and immunofluorescence analysis suggested that the expression of Schwann cell markers S-100, P75, and GFAP were significantly down-regulated in si-ERBB2 group than those in the control groups (Fig. 6a, b, d–f). ELISA assays also showed that interfering the expression of ERBB2 reduced the concentration of BDNF, NGF, and NT3 in the supernatant of SCLCs (Fig. 6c). The experiment showed that ERBB2 functioned a positive role in Schwann-like cell differentiation from hAMSCs.

It can be inferred from the above results that miR-146a-3p may inhibited the differentiation of hAMSCs into SCLCs by downregulating the expression of ERBB2.

## Discussion

Nowadays, autologous nerve transplantation is still the “golden standard” for repairing peripheral nerve defects, but there are several drawbacks such as limited sources of donors, residual donor site sensory dysfunction, and increased donor site trauma. For peripheral nerve growth, various drugs or nutrition factors are used to promote peripheral nerve repair and regeneration, but their clinical effects are limited (Menorca et al. 2013). Schwann cells, which are unique glial cells of the peripheral nervous system, can promote the repair and regeneration of peripheral nerve tissue. After a peripheral nerve injury, SCs phagocytize deformed axons and myelin sheath debris and rapidly proliferate to form Bungner bands, secrete neurotrophic factors, extracellular matrix, and adhesion molecules, guide axon growth direction, maintain neuronal vitality, and promote the peripheral reintroduction of the injured area (Stoll et al. 1989). However, a key problem has been encountered regarding the acquisition of highly purified SCs (Stefanescu et al. 2012).

Herein, we aimed to derive fate-committed SCLCs from hAMSCs such that sufficient numbers could be tapped on demand for transplantation to improve the prospects of post-traumatic re-growth and re-myelination of axons and recovery of function. With the use of stage-specific culture conditions, we achieved the goal of generating a high number of iSCs. The SCLCs displayed Schwann-like molecular phenotypic characteristics, such as the neurocyte-specific proteins S-100, P75, and GFAP and the neurotrophic factors BDNF, NGF, and NT3, as we expected. S-100, P75, and GFAP are neurocyte-specific markers that play an important role in peripheral nerve injury repair. Their expression indicates the formation of Schwann cells (Rutten et al. 2012). BDNF, NGF, and NT3 are members of the Schwann cell-derived neurotrophic factor family, which is closely related to the regeneration and differentiation of neurons. After peripheral nerve injury, NGF, BDNF, and NT3 are secreted and bind their high-affinity receptors trkA, trkB, and trkC and, then, activate the tyrosine kinase signaling system (Boyd and Gordon 2003). During repair, neurotrophic factors can avoid neuronal retrograde death after peripheral nerve injury, guide and promote axon regeneration, and promote myelin sheath formation. Our results indicated that SCLCs have the potential to function as mature human Schwann cells in the repair of peripheral nerve injury, which was consistent with the previous reports (Gon et al. 2018; Sanluis-Verdes et al. 2017).

Results from this study also showed that miR-146a-3p expression was clearly decreased at day 14 post-induction, approximately 0.4-fold less than prior to induction, further increased thereafter, and tended to stabilize at day 30. Together with a previous finding that miR-146a expression was down-regulated after acute intracerebral hemorrhage, we hypothesized that miR-146a plays a regulatory role in the process of hAMSC differentiation into Schwann cells (Zh et al. 2015).

The miR-146a gene is located in 5q34 and plays roles in diverse fields, including tumorigenesis, respiratory diseases pathogenesis, and immune reaction. MiRNA-146a was previously reported to be involved in cancer development and autoimmune disease (Bogunia-Kubik et al. 2016; Kotlarek et al. 2018; Marega et al. 2016). Recently, Hsa-miR-146a-3p was found be to a regulator of macrophage differentiation and M1/M2 polarized activation processes (Essandoh et al. 2016). Yi reported that hsa-miR-146a-3p negatively regulated the adipogenesis from human bone mesenchymal stem cells via targeting PLIN4 (Yi et al. 2019).

Results from this study showed that after interference with miR-146a-3p by using miR-146a-3p-inhibitor, induced SCLCs had an increased expression of Schwann cells-related neurotrophic factors while, after over-expression of miR-146a-3p, the differentiation capacity was significantly attenuated. It is noteworthy, this indicated that miR-146a-3p might function as a negative regulator not only on adiposegenes from hBMSCs as well as the not the differentiation of hAMSCs into SCLCs. Therefore, the role of miR-146a-3p in other MSC differentiation is worth further exploring. To investigate the mechanism by which miR-146a-3p regulated the differentiation of neural crest stem cells, we sought to find miR-146a-3p targets via databases. Results showed that a 7-mer-long sequence at the 3′UTR region of the ERBB2 mRNA was matched to miR-146-3p. ERRB2 encodes a member of the epidermal growth factor (EGF) receptor family of receptor tyrosine kinases. ERRB2 mainly exerted its regulatory function of specific receptor of neuregulin-1 (NRG1) by forming dimers with other EGF receptors. NRG1-ERRB2 signal pathways partake in the regulation of growth and development of glial cells, enteric neurons, and cardiomyocytes (Barrenschee et al. 2015; Grazette et al. 2004; Schmid et al. 2003).

In the peripheral nervous system, the number of SCs should correspond to the number of axons. The ERBB signal is clearly active during the development of SCs. It has been confirmed that inhibition of the ERBB tyrosine kinase receptor can inhibit the proliferation of SCs and promote their apoptosis, further affecting the migration of SCs to axonal terminals and axonal transplantation after nerve injury (Suzana et al. 2006). In addition, if a mutation occurs in ERBB, SCs’ transplantation to axons will be severely damaged (Lyons et al. 2005). Therefore, the ERBB signal is very important for the normal function of SCs. Thus, this study selected ERBB2 as a potential target of miR-146a-3p and investigated whether miR-146a-3p regulated the differentiation of amniotic mesenchymal stem cells by influencing the expression of ERBB2. We found that the expression of miR-146a-3p decreased and the expression of ERBB2 increased during the differentiation period. This suggested that ERBB2 was likely to be a down-stream target gene via which miR-146a-3p functions. Based on this finding, targeting miRNA-146a-3p expression during the differentiation of hAMSCs to SCLCs be a practical method to promote the production of SCLCs. However, further vivo experiments need to explore the efficacy and safety of SCLCs produced by this method to peripheral nerve injury.

## Conclusion

Taken together, this study demonstrated that human amniotic membrane stem cells could be differentiated into functional Schwann cell-like cells. By miRNA-seq, we identified that miRNAs were significantly associated with Schwann cell-like differentiation of hAMSCs. During the differential procedure, miR-146a-3p might play negative role through the down-regulating of the ERBB2 protein expression. The results might provide a new insight for batch acquisition of purified Schwann cells and further development into an autologous cell source for implantation as a safe therapy for nerve injuries or peripheral neuropathies.

## Abbreviations

hAMSCs: Human amniotic mesenchymal stem cells
BDNF: Brain-derived neurotrophic factor
GDNF: Glial cell-derived neurotrophic factor
NGF: Nerve growth factor
NT-3: Neurotrophin-3
DAPI: 4′,6-Diamidino-2-phenylindole
ARS: Alizarin red S
ERBB2: Erb-B2 receptor tyrosine kinase 2
SCLCs: Schwann-cell-like cells
S100: S100 calcium binding protein B
P75: TNF receptor superfamily member 1B
KEGG: Kyoto Encyclopedia of Genes and Genomes
GO: Gene Ontology
